# Effects of protein-enriched nutritional support on skeletal muscle mass and rehabilitative outcomes in brain tumor patients: a randomized controlled trial

**DOI:** 10.1038/s41598-024-63551-5

**Published:** 2024-06-05

**Authors:** Kye Hee Cho, Eun Young Han, Min Kyu Jung, Chang Moo Kang, Ji Cheol Shin, Sang Hee Im

**Affiliations:** 1 Department of Hospital Medicine, Yongin Severance Hospital, Yonsei University College of Medicine, Yongin, Republic of Korea; 2 Department of Rehabilitation Medicine, Jeju National University, Jeju, Republic of Korea; 3 Division of Medical Oncology, Department of Internal Medicine, Yonsei University College of Medicine, Seoul, Republic of Korea; 4 Division of Hepatobiliary and Pancreatic Surgery, Department of Surgery, Yonsei University College of Medicine, Seoul, Republic of Korea; 5 Department and Research Institute of Rehabilitation Medicine, Severance Rehabilitation Hospital, Yonsei University College of Medicine, 50-1 Yonsei-Ro, Seodaemun-Gu, Seoul, Republic of Korea

**Keywords:** Protein supplementation, Brain tumor, Muscle mass, Strength, Malnutrition, Function, Rehabilitation, CNS cancer, Malnutrition, CNS cancer

## Abstract

Patients with brain tumors require extensive and prolonged rehabilitation efforts as they suffer from lesion-induced motor weakness as well as treatment-related side effects, often leading to a significant decline in function. Protein supplements have shown positive effects on promoting muscle strength and physical performance in various tumor etiologies. However, reports on their effects specifically in brain tumor patients remain scarce. This study aims to investigate the feasibility and efficacy of protein supplements in enhancing rehabilitative outcomes via muscle strengthening and functional gain in brain tumor patients with neurological demise. Sixty brain tumor patients were randomly assigned to either a protein supplement or a control group, receiving either protein supplements or a placebo for 6 weeks, in conjunction with conventional rehabilitation therapy. Assessments before and after the intervention included laboratory tests, anthropometric measures using bioimpedance analysis, and functional assessments, which included the MMSE, the modified Barthel Index, the Beck Depression Inventory, the Brief Fatigue Inventory, the Timed Up and Go test, the 6-min walk test, the isokinetic quadriceps muscle strength test, and the handgrip power. After the intervention, the levels of serum hemoglobin, protein, albumin, and C-reactive protein were improved in both groups, however, the change was significant only in the protein group. The muscle strength was enhanced in both groups, however, the significant increase in pinch grasp power was only noted in the protein group (P < 0.05). The distance on 6MWT was also significantly extended at follow-up in the protein group (P < 0.01). In the subgroup analysis according to nutritional status, the moderate malnutrition group showed greater augmentation of muscle mass than those with adequate nutrition (P < 0.05). Interestingly, the amelioration of malnutrition was observed only the in protein group. This study using protein supplements to promote the rehabilitative potential of brain tumor patients revealed a significant effect on improving hemodynamic nutritional indices, muscle power reimbursement, and functional improvement, especially in malnourished patients. The safety and feasibility of protein supplements in brain tumor patients were affirmative in this study. Further studies with more patients may help confirm the secondary functional gain resulting from increased muscle power.

Trial registration: This study was retrospectively registered in the Clinical Research Information Service, CRIS no. KCT0009113 on Jan 12, 2024.

## Introduction

Patients with brain tumors face significant challenges due to neurological impairments caused by the tumor and side effects of treatments. These difficulties often result in motor deficits such as weakness, incoordination, and imbalance, limiting physical activity. Additionally, patients may experience loss of appetite and fatigue, further compromising nutritional intake and overall well-being^1–3^. Dietary interventions have been recognized in cancer care for their role in reducing morbidity, treatment-related toxicities, and enhancing quality of life^4–6^. Protein supplements, in particular, have been effective in restoring muscle health in malnourished advanced cancer patients^7^. However, the specific impact of nutritional strategies on the physical and functional recovery of brain tumor patients remains underexplored, despite evidence linking dietary modifications to improved survival outcomes^8^. Therefore, this study aims to examine the benefits of protein-enriched diets in enhancing functional outcomes, with a particular focus on muscle strengthening, in brain tumor patients.

## Methods

Brain tumor patients referred to the Department of Rehabilitation Medicine of a tertiary university hospital for rehabilitation therapy were recruited for this study between Dec 2020 and Jun 2022. A total of 179 brain tumor patients were assessed for eligibility. Inclusion criteria were (1) histologically confirmed diagnosis of brain tumor, (2) limited function and mobility due to weakness related to the tumor, and (3) agreeable to participate in in-hospital rehabilitation therapy. Exclusion criteria were (1) ages younger than 18 years or older than 80 years old, (2) any history of the following event within 3 months; myocardial infarction, unstable angina, coronary angioplasty, cardiac failure NYHA class III or IV, stroke or transient ischemic attack, (3) active infection or sepsis, (4) deep vein thrombosis within 4 weeks, (5) pregnant or breastfeeding, (6) hepatic failure, abnormal liver function test, (7) diagnosed diabetes mellitus, (8) on parenteral nutrition for electrolyte imbalance.

**Figure 1:**
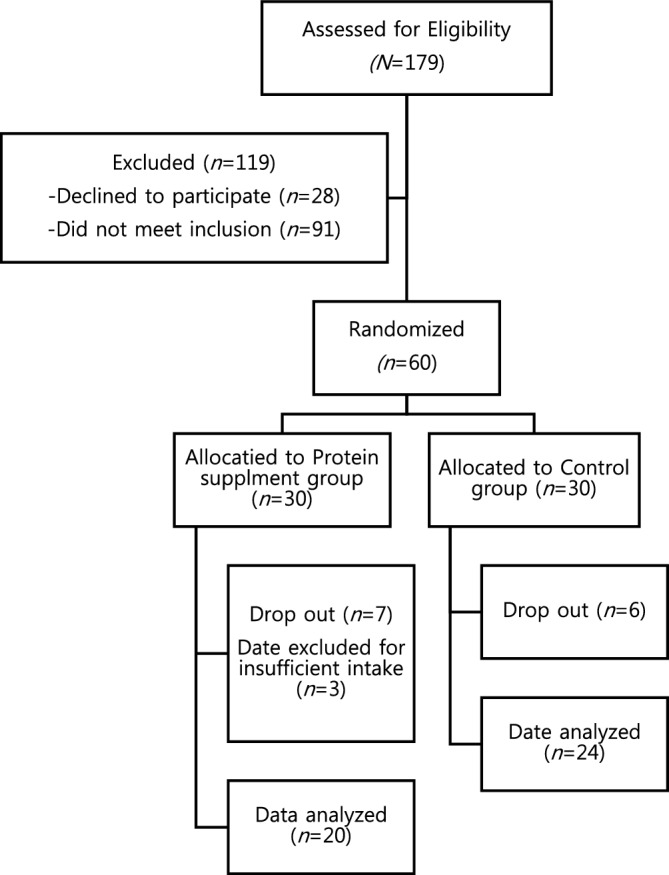
Flow chart of study process.

After screening for exclusion criteria, 88 patients were informed about this study, and 60 patients agreed to participate and signed the informed consent form. They were randomly assigned to either the protein group or the control group. Each group was blinded to receive a respective protein supplement or placebo, 18 g each, every day for six weeks. During follow-up, 13 patients dropped out; four patients could not fulfill the study duration by early discharge, four patients for deconditioning, three patients for abdominal discomfort, and two developed uncontrolled serum glucose levels requiring treatment. Among 47 patients who completed the study, 44 patients were included in the analysis excluding three patients who consumed less than 75% of protein supplementation (Fig. 1).

A professional nutritionist blinded to the allocation assessed the basal nutritional habits including the history of smoking, alcohol, use of alternative medicine, and dietary supplements. The amount of energy consumption for each patient was recorded and calculated using CAN-pro 5.0 (Computer Aided Nutritional Analysis Program for Professionals, The Korean Nutrition Society) based on the daily diet log of each patient. Regardless of the group, the amount of daily consumed protein was 1.2–1.3 g/kg based on the nutritional evaluation of each participant. The level of malnutrition was assessed based on the American Society for Parenteral and Enteral Nutrition Classification (ASPEN)^9^. The Scored Patient-Generated Subjective Global Assessment was used for nutritional status assessments^10^.

### Conventional rehabilitation therapy

In-patient rehabilitation therapy for 6 weeks was provided to all patients. Rehabilitation therapy consisted of physical therapy, occupation therapy, dysphagia therapy, speech therapy, and cognitive rehabilitation therapy depending on the individual need. Daily physical therapy was performed by experienced physical therapists for brain tumor. Depending on the individual functional level, training tasks consisted of rolling, sitting balance training, transfer, passive and active range of motion exercise, active or assistive strengthening exercises, and assisted standing on a tilt table while monitoring autonomic nervous system symptoms, such as faintness and pain. When capable, patients were also engaged in active or assisted cycling, assisted gait training, or self-directed walking. Physical therapy was conducted at least 30 min a session, twice a day for more than 5 days a week.

Occupational therapy consisted of assistive upper limb stretching exercises to prevent contractures and maintain muscle strength and endurance and task-oriented therapy for activities of daily living. The proper use of an environmental control unit, orthotics, and splints for assisting hand function, transfer, and wheelchair manipulation was also taught after wheelchair adjustments were made.

### Protein supplement or placebo

The protein mixture power (Yonsei Health Care Protein) product, a 9 g powder pack developed by Yonsei University Dairy, Chungcheongnam-do, Korea was provided as a protein supplement. A pack of the protein powder was to be mixed with 100 ccs of water for use. Two packs were given to the protein group each day, to be consumed either as one pack twice a day or two packs at once. The control group received placebo packs, identical in design to the protein powder packs, every day during the study.

Each pack of powder includes 9 g of proteins (calcium casein, milk concentrate, whey protein, soybean protein, Faba Bean protein, pea protein, and rice protein), prebiotics (Fructo-oligosaccharides) for indigestion, Vitamins/Minerals (Zinc, Magnesium, Calcium, Niacin, Pantothenic Acid, Folic Acid, Biotin, Vitamin B1/B2/C/B6/D/E), and sugar alternatives (Palatinose, Fructose). The placebo supplement consisted of the same amount of carbohydrate instead of protein, which may influence blood sugar, thus, patients with diabetes were excluded.

### Assessments

Laboratory tests, bioelectrical impedance analysis (BIA, InBODY S10, South Korea), and anthropometric indices were conducted before and after the intervention. Additionally, we assessed neurocognitive function, physical function, level of independence in daily activities, and cancer-specific functional assessments. Medical technologists and physical therapists blinded to the randomization carried out all evaluations.

Laboratory data included white blood cell count, absolute neutrophil count, hemoglobin, hematocrit, neutrophil–lymphocyte ratio, and chemistry including the level of blood urea nitrogen, creatinine, estimated glomerular filtration rate, aspartate aminotransferase, alanine aminotransferase, albumin, total cholesterol, and C-reactive protein (CRP). BIA data include muscle mass, skeletal muscle mass, and percentage body fat.

The muscle strength of the quadriceps, handgrip, and pinch grip were assessed. Isokinetic quadriceps peak torque at 60 and 120 degrees for each leg was measured in a seated position with a Cybex NORM dynamometer (Cybex Inc., USA). The mean data of 5 repetitions was collected. Handgrip strength including grasp power and lateral pinch power was assessed by a digital grip dynamometer (Jamar Tech., USA). In consideration of weakness stemming from brain lesions, the assessment of strength used the mean value of the bilateral limbs for the analysis.

Mini-Mental State Examination (MMSE) was used as a brain lesion-related neurocognitive functional assessment. It is an objective assessment of a 30-point questionnaire that screens for cognitive decline in domains of arithmetic, memory, and orientation^11^. The modified Barthel Index (MBI) was used to assess the performance of daily activities of living^12^ which impacts the quality of life.

The assessments of physical function were for mobility, speed, and endurance including Timed Up and Go (TUG), 6-minute walk test (6MWT), and 10-meter walk test. The TUG test assesses mobility and balance by measuring the time it takes for a person to rise from a chair, walk three meters, turn around, walk back to the chair, and sit down again^13^. The 6MWT measures the distance a person can walk in 6 minutes at their own pace to assess exercise capacity and endurance^14^. A 10-meter walk test was used to assess the gait speed and mobility^15^.

As cancer-specific functional assessments, the Beck Depression Inventory (BDI) and Brief Fatigue Inventory (BFI) were utilized. Both the BDI^16,17^ and BFI^18^ are well-validated, self-report questionnaires designed to quantify the degree of symptoms of depression and fatigue, respectively. Higher scores indicate greater severity of the symptoms.

For the estimation of trial feasibility, a formal sample size calculation was not necessary^19^. However, based on a previous study on the feasibility of exercise and nutrition-based rehabilitation for cancer patients^6^, 40 patients were estimated as the target size. In consideration of the physical condition of brain tumor participants under weakness, we estimated 50% drop out. Approximately 80 participants would have been needed. T-test was used to compare both groups, while the Mann–Whitney *U* test was for the data not satisfying normal distribution. A paired T-test was used to compare the initial and follow-up assessments of each group. Mann–Whitney test was used for the subgroup analysis based on the nutritional status. This study was conducted following the recommendations of the institutional review board of Yonsei University Health System, which also approved the protocol used in this study (ethical approval number: 4-2020-0584). All study methods were executed in accordance with the Declaration of Helsinki and the CONSORT reporting guidelines^20^.

## Results

Baseline characteristics of the protein supplement (n = 20) and control (n = 24) groups were not different between groups, except for quadriceps peak torque (Tables 1, 2, 3). All participants had histologically confirmed brain tumors. Two participants had metastatic brain tumors: one from breast cancer, the other from lung cancer. Tumors originating other than meninges and glial cells included hemangioblastoma, craniopharyngioma, and neurocytoma. Molecular markers, histologically confirmed, did not show difference between groups (Table 1). The daily amount of protein consumed at baseline and protein consumption percentage relative to minimal requirements were not different between groups prior to the study. The compliance with protein supplement usage tended to be higher in the protein group than in the control group (P = 0.052). There was no significant different in the protein and control groups in muscle power and mass gain as well as function after the intervention.

**Table 1:** Baseline characteristics of study populations.

	All (n = 44)	Protein group (n = 20)	Control group (n = 24)	P value
Age (years, range)	55.2 ± 13.4	51.4 (21–73)	58.0 (39–79)	0.116
Male/female	20/25	10/10	10/14	0.402
Affected side (hemi/bilateral)	37/7	19/1	18/6	0.079
Onset to study (months)	13.1 ± 29.4	13.6 ± 27.8	12.8 ± 31.9	0.930
Baseline protein consumption (g)	69.7 ± 21.9	74.0 ± 22.3	66.1 ± 21.3	0.237
Height (cm)	163.8 ± 8.5	164.3 ± 7.3	163.4 ± 9.5	0.722
Weight (kg)	60.4 ± 10.1	61.0 ± 9.5	59.9 ± 10.7	0.730
BMI(kg/m^2^)	22.5 ± 3.2	22.6 ± 3.4	22.3 ± 3.0	0.534
MMSE^a^	19.8 ± 9.9	20.6 ± 9.2	19.0 ± 10.5	0.967
Types of brain tumor				
Meningioma	5	2	3	
Glioma (grade I, II)	6	3	3	
High-grade glioma	28	13	15	
Glioblastoma	19	9	10	
Anaplastic astrocytoma	9	4	5	
Molecular markers				
IDH1/2 mutation ±	9/23	5/12	4/11	0.589
MGMT methylation ±	15/15	7/8	8/7	0.726
1p/19q intact/co-deletion	19/6	10/3	9/3	0.637
Metastatic tumors	2	0	2	
Others	3	2	1	
Combined therapy				
Chemotherapy only	2	0	2	0.292
Radiation therapy only	12	6	6	0.486
CCRT	21	11	10	0.282
ASPEN nutrition state				
Well-nourished	15	7	8	0.769
Mild malnutrition	14	5	9	0.769
Moderate malnutrition	15	8	7	0.769
Comorbidity				
DM	0	0	0	
Hypertension	13	7	6	0.347
Dyslipidemia	2	1	1	0.708

Values are mean ± standard deviation or number.

^a^Mann–Whitney *U* test for not satisfying Kolmogorov–Smirnov test otherwise T-test.

*BMI* Body Mass Index, *MMSE* Mini Mental Status Examination, *BDI* Beck Depression Inventory, *BFI* Brief Fatigue Inventory, *MBI* modified Bathel Index, *IDH 1/2* Isocitrate dehydrogenase 1 or 2, *MGMT* O(6)-methylguanine-DNA methyltransferase, *CCRT* Concurrent Chemoradiotherapy, *ASPEN* American Society for Parenteral and Enteral Nutrition Classification, *DM* diabetes mellius.

*Others* hemangioblastoma, craniopharyngioma, neurocytoma.

**Table 2:** Group differences after protein supplement in anthropometric and laboratory results.

	All (n = 44)		Protein (n = 20)		Control (n = 24)	
	Initial	Follow-up	Initial	Follow-up	Initial	Follow-up
Weight (kg)	60.4 ± 10.1	61.1 ± 9.3	61.0 ± 9.5	61.8 ± 8.8	59.9 ± 10.7	60.5 ± 9.8
BMI (kg/m^2^)	22.5 ± 3.2	22.8 ± 2.9	22.6 ± 3.4	22.9 ± 3.0	22.3 ± 3.0	22.7 ± 2.9
SMM (kg)	22.6 ± 4.8	23.2 ± 4.7	22.7 ± 4.0	23.7 ± 4.0	22.5 ± 5.4	22.7 ± 5.3
SMI (kg/m^2^)	8.4 ± 1.3	8.6 ± 1.2	8.4 ± 1.2	8.8 ± 1.0	8.3 ± 1.3	8.4 ± 1.3
FFM (kg)	42.2 ± 7.9	43.1 ± 7.7	42.2 ± 6.7	44.0 ± 6.5	42.1 ± 9.0	42.3 ± 8.7
FFMI (kg/m^2^)	15.6 ± 2.0	16.0 ± 1.8	15.6 ± 2.1	16.2 ± 1.6	15.6 ± 2.1	15.7 ± 2.0
BFM (kg)	18.2 ± 6.1	17.9 ± 5.7	18.8 ± 7.3	17.8 ± 7.1	17.7 ± 5.0	18.1 ± 4.3
FMI (kg/m^2^)	6.8 ± 2.4	6.8 ± 2.4	7.0 ± 2.8	6.7 ± 2.9	6.7 ± 2.1	6.9 ± 1.9
Body fat (%)	29.9 ± 8.0	29.3 ± 8.1	30.2 ± 9.1	28.2 ± 9.6	29.7 ± 7.1	30.2 ± 6.7
WBC (10^3^cell/L)	5.8 ± 1.8	5.5 ± 2.3	5.7 ± 1.8	5.5 ± 2.0	5.9 ± 1.8	5.5 ± 2.7
TLC (10^3^cells/L)	1.4 ± 5.7	1.3 ± 6.7	1.4 ± 4.8	1.2 ± 5.3	1.4 ± 6.3	1.3 ± 7.8
Hemoglobin (g/dL)	11.2 ± 1.4	12.0 ± 1.3^††^	11.0 ± 1.2	12.0 ± 1.5^†^	11.4 ± 1.5	12.0 ± 1.2
Protein (g/dL)	5.9 ± 0.5	6.2 ± 0.4^†^	5.9 ± 0.5	6.3 ± 0.5^††^	6.0 ± 0.6	6.1 ± 0.3
Albumin (g/dL)	3.7 ± 0.5	4.0 ± 0.3^††^	3.8 ± 0.3	4.0 ± 0.3^††^	3.7 ± 0.6	3.9 ± 0.2
Total cholesterol (mg/dL)	187.9 ± 47.2	173.8 ± 38.0	190.0 ± 46.9	173.4 ± 40.1	186.1 ± 48.4	174.2 ± 37.4
CRP (mg/L)	5.3 ± 7.5	3.7 ± 6.9^††^	5.1 ± 5.4	2.7 ± 3.7^†^	5.4 ± 8.9	4.6 ± 8.7

Values are mean ± standard deviation. Values are mean ± standard deviation.

^†^P < 0.05, ^††^P < 0.01, Paired t-test between initial and follow-up assessments.

*BMI* body mass index, *SMM* skeletal muscle mass, *SMI* skeletal muscle mass index, *FFM* fat free mass, *FFMI* fat free mass index, *BFM* body fat mass, *FMI* fat mass index, *WBC* white blood cell, *TLC* total lymphocyte count, *CRP* c-reactive protein.

**Table 3:** Group differences after protein supplement in cognitive and physical function.

	All (n = 44)		Protein (n = 20)		Control (n = 24)	
	Initial	Follow-up	Initial	Follow-up	Initial	Follow-up
MMSE	20.0 ± 9.9	22.8 ± 8.6^††^	20.6 ± 9.2	22.5 ± 8.9^††^	19.0 ± 10.5	23.0 ± 8.5^††^
BDI	15.4 ± 11.0	13.0 ± 9.8	13.6 ± 10.2	12.9 ± 9.2	15.7 ± 10.7	13.0 ± 10.5
BFI	4.0 ± 2.3	3.9 ± 1.9	3.8 ± 2.3	4.0 ± 2.1	4.2 ± 2.2	3.8 ± 1.8
BBS	20.1 ± 16.5	33.1 ± 17.1^††^	19.5 ± 15.8	34.5 ± 17.6^††^	20.6 ± 17.4	31.9 ± 16.9^††^
MBI	42.7 ± 26.9	59.9 ± 30.1^††^	45.5 ± 25.9	62.9 ± 26.7^††^	40.5 ± 28.0	57.5 ± 33.1^††^
6MWT (m)^a^	67.0 ± 117.1	124.0 ± 151.6^††^	69.4 ± 135.3	122.0 ± 150.9^††^	65.1 ± 102.5	125.7 ± 155.4
10MWT (s)	16.4 ± 9.6	15.5 ± 9.4	15.9 ± 13.6	15.2 ± 14.0	18.2 ± 4.8	16.5 ± 13.5
TUG (s)	21.0 ± 10.1	18.9 ± 12.1	18.0 ± 11.6	20.9 ± 14.8	24.4 ± 8.9	17.2 ± 9.6
Grip power (kg)	15.6 ± 9.4	17.1 ± 9.0^††^	16.8 ± 10.5	18.1 ± 10.4^††^	14.5 ± 8.4	16.3 ± 7.8^††^
Pinch power (kg)	9.1 ± 4.3	10.7 ± 4.7^††^	9.0 ± 4.7	12.0 ± 4.6^††^	8.5 ± 4.1	9.8 ± 4.5
Quad torque 60°(Nm)*	22.0 ± 14.8	37.3 ± 26.0^††^	27.9 ± 15.9	42.1 ± 30.2^††^	17.2 ± 12.2	33.3 ± 21.7^††^
Quad torque 120°(Nm)*	15.5 ± 10.1	28.1 ± 21.5^††^	19.0 ± 9.8	31.8 ± 26.1^††^	12.6 ± 9.5	24.9 ± 16.8^††^

Values are mean ± standard deviation.

*P < 0.05, T-test between protein and control groups for the initial assessment.

^†^P < 0.05, ^††^P < 0.01, Paired t-test between initial and follow-up assessments.

^a^Wilcoxon sign test for not satisfying Kolmogorov–Smirnov test.

*MMSE* Mini Mental Status Examination, *BBS* Berg Balance Scale, *MBI* modified Barthel Index, *BFI* Brief Fatigue Inventory, *BDI* Beck Depression Inventory, *6MWT* 6-Minute Walk Test, *10MWT* 10-Meter Walk Test, *TUG* Timed Up and Go, *Quad torque 60° and 120°* quadriceps muscle peak torque at 60° and 120°.

After the 6-week intervention period, all patients of both groups showed improvements in the anthropometric measures. However, change of anthropometric indices of BIA showed different pattern between groups. The skeletal muscle mass and fat-free mass tended to increase in both groups, however, the fat contents including body fat mass, fat mass index, and body fat percentage tended to decrease in the protein group and increase in the control group (Table 2).

Laboratory results including the level of serum hemoglobin, protein, albumin, and CRP were also improved in all patients at follow-up. However, the improvements in hemoglobin, protein, albumin, and CRP were statistically significant only in the protein group (Table 2, P < 0.05).

Functional assessments, MMSE, Berg balance scale (BBS), MBI, grip power, and quadriceps muscle peak torque at 60 and 120 degrees were improved in both groups compared to the initial assessment (P < 0.01). However, the BDI and BFI scores showed no significant differences in the assessment before and after. In between group comparisons, significant improvement was noted specifically in 6MWT and pinch grasp power and it was noted only in the protein group (P < 0.05) (Table 3).

**Figure 2:**
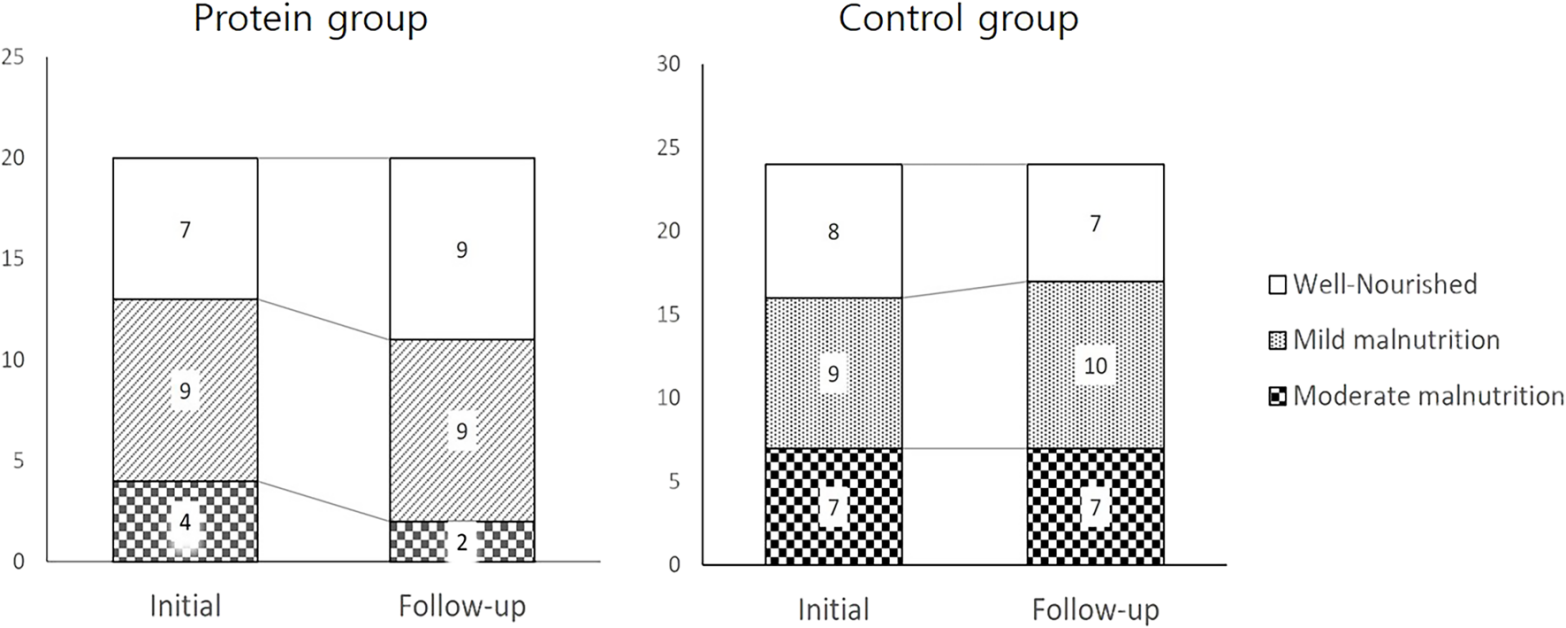
Change of malnutrition status defined by ASPEN guideline between groups. *ASPEN* American Society for Parenteral and Enteral Nutrition Classification.

The state of malnutrition assessed by ASPEN guidelines showed a different tendency of change between groups after the protein supplementation. At follow-up, 65% (n = 13) of malnutrition patients in the protein group decreased to 55% (n = 11). Notably, the number of moderate malnutrition patients decreased from 4 to 2, and the resolution of malnutrition was observed only in the protein group. On the contrary, the overall number of malnutrition patients increased from 16 to 17 in the control group. The number of patients with no malnutrition only increased in the protein group (Fig. 2).

**Figure 3:**
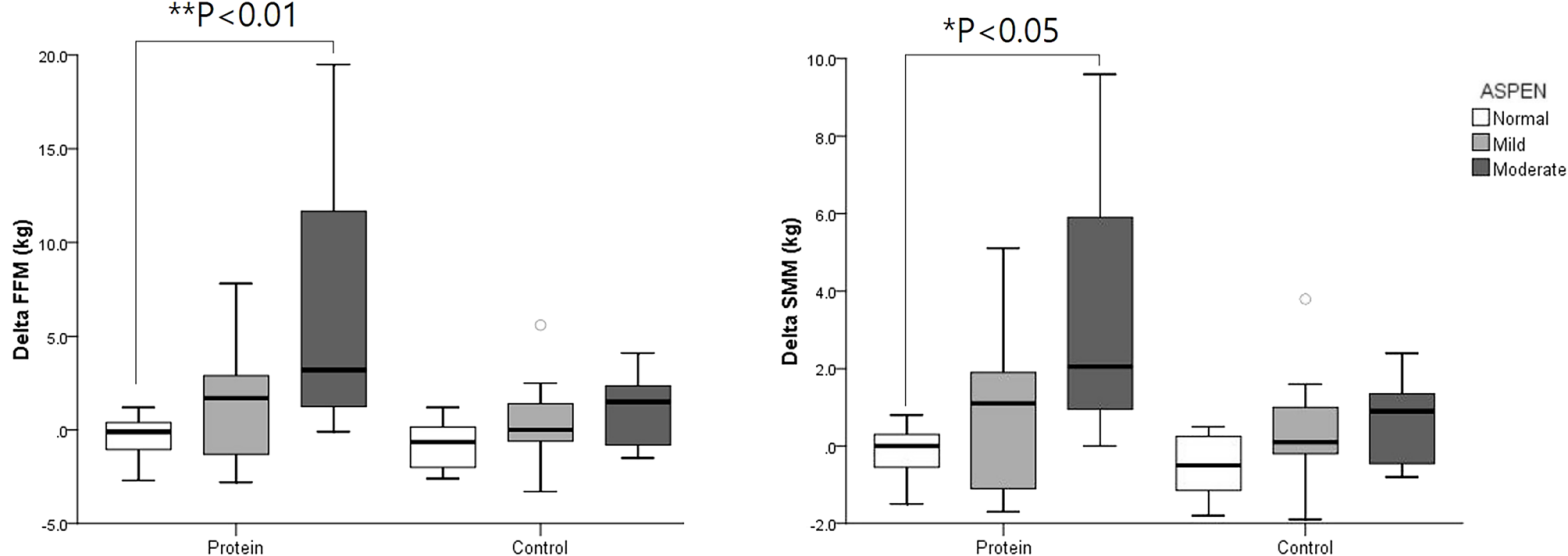
Change of fat-free mass and skeletal muscle mass depending on nutritional state in protein and control groups. *FFM* Fat-free mass, *SMM* Skeletal muscle mass, *ASPEN* American Society for Parenteral and Enteral Nutrition Classification.

**Figure 4:**
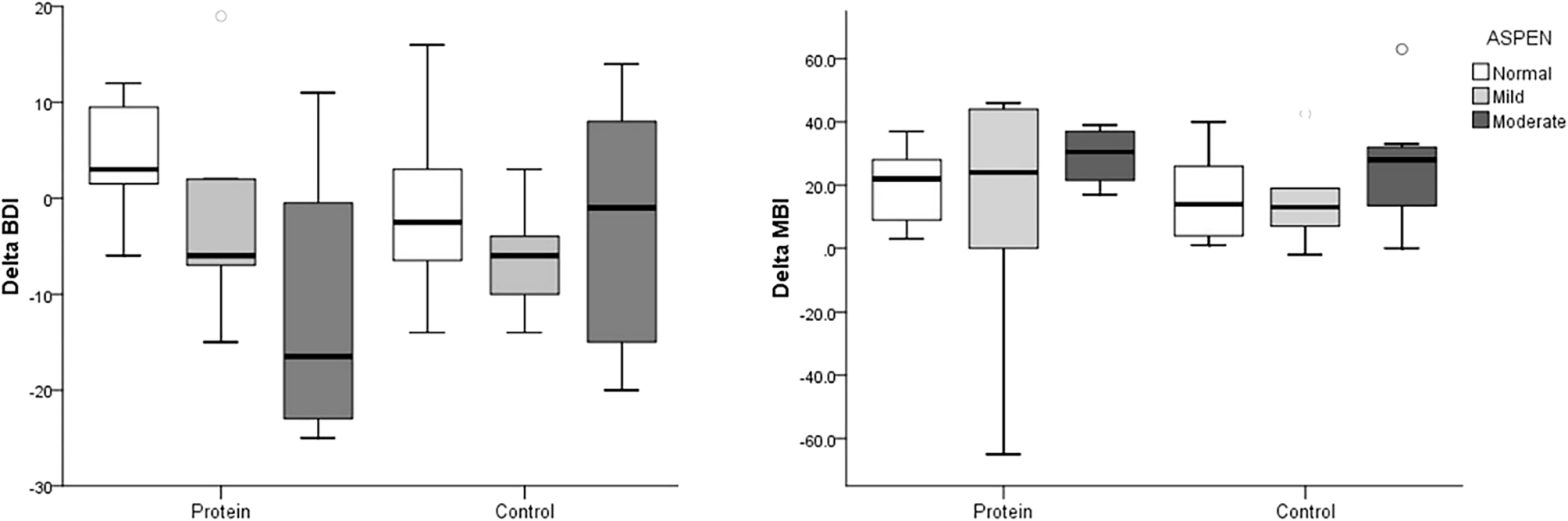
Change of functional assessment in BDI and MBI depending on the malnutrition state in protein and control groups. *BDI* Beck Depression Inventory, *MBI* modified Barthel Index, *ASPEN* American Society for Parenteral and Enteral Nutrition Classification.

Sub-group analysis, following ASPEN guidelines for malnutrition classification, revealed significant differences in body composition changes post-intervention, particularly in fat-free mass and skeletal muscle mass. Significantly greater increases in skeletal muscle mass, body cell mass, and fat-free mass were observed in the moderately malnourished group compared to those with adequate nutrition within the protein group (P < 0.05; Table 4, Fig. 3). The Nutritional state significantly influenced the percentage change in BBS within the protein group, as determined by the Kruskal Wallis test (P < 0.05). However, there were no significant differences in depression and independence levels, as assessed by the BDI and MBI, between groups based on nutritional state (Fig. 4).

**Table 4:** Change of body content and strength according to nutritional status.

Malnutrition state	Protein			Control		
	None (n = 7)	Mild (n = 9)	Moderate (n = 4)	None (n = 8)	Mild (n = 9)	Moderate (n = 7)
∆Skeletal muscle mass (kg)	− 0.2 ± 0.8 (− 0.9)	0.9 ± 2.1 (4.0)	3.4 ± 4.2 (19.6)*	− 0.5 ± 0.9 (− 1.8)	0.4 ± 1.7 (2.0)	0.6 ± 1.2 (3.6)
∆Body cell mass (kg)	− 0.2 ± 0.9 (− 0.8)	0.9 ± 2.3 (3.5)	3.7 ± 4.6 (17.5)*	− 0.6 ± 0.9 (− 1.6)	0.5 ± 1.8 (1.9)	0.7 ± 1.3 (3.2)
∆Fat-free mass (kg)	− 0.4 ± 1.3 (− 1.0)	1.4 ± 3.2 (3.3)	3.4 ± 4.2 (20.8)**	− 0.8 ± 1.4 (− 1.6)	0.5 ± 2.7 (1.2)	1.0 ± 2.1 (3.0)
∆Body fat mass	0.3 ± 1.8 (1.0)	− 1.3 ± 3.4 (− 9.6)	− 2.6 ± 8.9 (− 2.2)	1.1 ± 3.0 (9.1)	− 1.0 ± 2.2 (− 4.1)	1.4 ± 4.1 (14.2)
∆Body fat (%)	0.6 ± 0.8	− 2.2 ± 6.1	− 5.8 ± 14.3	1.6 ± 3.3	− 1.3 ± 3.4	1.5 ± 5.1
∆Grip power (kg)	4.2 ± 8.2 (12.2)	5.8 ± 13.4 (16.8)	9.1 ± 2.5 (95.7)	3.1 ± 9.0 (8.8)	2.6 ± 6.8 (11.4)	3.4 ± 4.3 (36.9)
∆Pinch power (kg)	1.8 ± 3.4 (22.4)	1.8 ± 4.4 (21.6)	3.8 ± 1.5 (109.3)	0.8 ± 3.5 (14.5)	1.4 ± 3.2 (18.9)	1.2 ± 2.3 (26.0)
∆Quad torque 60°(Nm)	2.7 ± 12.6 (10.1)	8.1 ± 16.6 (42.5)	12.3 ± 6.6 (91.8)	8.8 ± 7.5 (67.5)	9.3 ± 7.9 (41.9)	5.3 ± 6.0 (145.7)
∆Quad torque 120°(Nm)	2.1 ± 10.2 (8.3)	7.1 ± 15.3 (45.5)	12.3 ± 5.8 (112.3)	8.3 ± 7.4 (80.1)	6.1 ± 5.1 (38.9)	3.4 ± 4.5 (130.5)

Values are mean ± standard error.

*P < 0.05, **P < 0.01, Mann–Whitney *U* test between the none and moderate malnutrition subgroups.

∆, change between the initial and follow-up values.

Correlation analysis indicated that, within the protein group, significant positive relationships were observed between changes in the BBS and both quadriceps peak torque at 60 and 120 degrees/sec, and muscle mass in SMM and FFM (Table 5). The correlation between improvements in muscle strength and mass with increased functional independence, as assessed by the MBI, was also significant only in the protein group (Table 5). The improvement in balance significantly correlated with enhanced functional independence, with substantial correlations noted between BBS and MBI changes in both the protein (r = 0.725, P < 0.001) and control groups (r = 0.562, P < 0.01). The correlation between changes in the BDI and BFI was also significant in both groups (P < 0.01).

**Table 5:** Correlation between functional assessments and muscle mass and strength improvements according to groups.

	∆BBS		∆MBI	
	Protein	Control	Protein	Control
∆Skeletal muscle mass (kg)	0.589**	0.260	0.558*	0.096
∆Fat-free mass (kg)	0.601**	0.304	0.566**	0.083
∆Grasp power (kg)	0.242	0.199	0.686**	0.225
∆Pinch power (kg)	0.355	0.212	0.557*	− 0.151
∆Quad torque 60°(Nm)	0.523*	0.289	0.665**	0.099
∆Quad torque 120°(Nm)	0.506*	0.340	0.602**	0.035

Values are Spearman's ρ.

*P < 0.05, **P < 0.01, Spearman correlation. ∆, change between the initial and follow-up values.

There was no significant change in the level of BUN, Creatinine, aspartate aminotransferase, alanine aminotransferase, and total cholesterol after the protein supplement. The protein supplement did not cause any gastrointestinal or alimentary side effects in need of medical attention except for a patient who withdrew participation due to abdominal discomfort after the protein supplement.

## Discussion

The nutritional impact on the physiologic and functional outcome in brain tumor patients was evident after the short intervention of a 6-week protein surplus. A significant laboratory, anthropometric, and functional improvement was evident in the protein group after the intervention. This is the direct evidence of protein supplements showing a positive effect on physiological and functional improvement leading to better rehabilitative outcomes in brain tumor patients.

In the protein group, the inflammatory marker CRP was reduced consistent with previous studies on higher-quality diets. The quality of a diet is assessed by the Healthy Eating Index (HEI)^21^, which is a sum of the nutritional components of a diet. A higher HEI score implies that the diet aligns with dietary guidelines^22^. The diets with higher adherence to the HEI were associated with low inflammatory potential in breast tumors^23^ and lower CRP levels in various tumor patients^24^. The positive effects of protein supplementation could be attributed to the anti-inflammatory effect, as protein-rich diets such as total protein foods, seafood and plant proteins, and greens and beans yield higher points in HEI^22^. However, significant laboratory changes compared to the baseline were observed only in CRP, hemoglobin, protein, and albumin, likely due to the short-term follow-up period of 6 weeks in this study. These findings are expected to have long-term implications. Immunity-related markers, including total lymphocyte count (TLC) and the ratio between absolute neutrophil count and absolute lymphocyte count, did not exhibit any significant differences between groups. Although TLC is a component of the prognostic nutritional index, a prognostic parameter related to poor outcomes in brain tumors^25^, the relatively normal initial TLC levels of participants may have caused the indifference in our study.

In conjunction with the laboratory improvement, attenuation of FFM reduction and higher fat mass loss occurred, specifically in the protein group. It is consistent with a previous report on higher protein supplementation protecting FFM loss during energy deficit^26^. Given the circumstances of the post-tumor treatment, they may be experiencing an anabolic muscle response after protein supplement similar to higher protein intake during energy deficit, leading to enhanced anabolic responses. In fact, protein supplementation had more pronounced effects on muscle strength and mass in the presence of poor oral intake in various tumors^27^. Furthermore, in the subgroup analysis according to nutritional state based on ASPEN classification, the muscle mass gain was significantly greater in those under moderate malnutrition than in those with adequate nutrition. The enhancement of muscle power also showed the tendency to be greater in the malnutrition group. In addition, malnutrition was ameliorated only in the protein group despite the short duration of six weeks for supplementation. Accordingly, patients with malnutrition may require more protein supplements, and further research is needed to determine the appropriate dosage. Overall, these findings support the use of protein supplements in brain tumor patients.

The amount of protein supplement given in this study was 18 g which is above what is recommended by nutritional evaluation. The acceptable macronutrient distribution range for protein in adults has been indicated as between the RDA (Recommended Dietary Allowance) and the upper limit associated with the capability of urea synthesis (i.e., 0.8–3.5 g/kg/day)^28^. Consuming higher than the RDA of protein for essential amino acids to achieve high muscle protein synthesis is suggested for muscle mass preservation^29^. Essential amino acids activate the mammalian target of rapamycin (mTOR) signaling pathway in muscle cells which integrates signals that regulate cell growth and metabolism^30^. It is estimated that a 100% increase in peripheral essential amino acids concentration corresponded to upto 34% increase of muscle protein fractional synthesis rate^31^, meaning large increase of peripheral amino acids required to drive robust increase in muscle protein synthesis. In healthy young subjects confined to bed rest, both muscle mass and strength were improved by increased availability of amino acids, even in the complete absence of activity^32^. Similarly, leucine-rich amino acids supplement resulted in significantly increased lean body mass and muscle strength even in the absence of exercise^33^. Hence, a high protein supplement itself may also have the potency to promote muscle mass and strength in brain tumor patients, provided that a high protein intake is not contraindicated.

Initially, the isokinetic peak torque of the quadriceps was different between the two groups. However, these differences disappeared after the intervention, even though both groups exhibited increased power at the follow-up. This lack of group difference at post-intervention might be attributed to the low initial peak torque due to the hemi- or quadriplegic conditions of patients. The isokinetic quadriceps muscle strength of participants was much lower compared to that of age-related peers (mean quadriceps peak torque at 60 degrees 22.0 vs. 71.6 Nm)^34^; however, muscle mass was relatively well-preserved in this study, and none of the participants were sarcopenic. It is important to note that low muscle strength can also occur even without low muscle mass^35^. According to research, when tumor-related muscle loss is present, the correlation between muscle mass and strength is fair^36^. Considering the overall positive trend in the muscle mass change, albeit without significant differences between groups, muscle mass may not have played a critical role in the functional improvement observed in the protein group.

The investigation of the effect of protein supplements in enhancing muscle strength and functional improvement yielded significant findings. The protein group showed significant improvement in pinch grasp power and the distance on 6MWT following the intervention. Handgrip is an independent marker of biological, functional, and quality of life associated with survival in advanced tumor patients^37^. Interestingly, endurance gain via 6MWT was noted after the 6-week intervention, although the results on TUG or 10MWT were not significant. The duration of the intervention may not have been enough to promote functional mobility or speed. Also, the initial performance of participants, which was considerably below typical references ranges, may have hindered significant change. The initial mean TUG of participants was 21.0 ± 10.1 s which is twice slower than the normal limits of up to 10 s, which is expected in healthy older adults^38^. The mean time needed for walking 10 m was 16.4 ± 9.6 s twice slower than the age-matched gait speed of 8.6 s^15^. The performance observed in 6MWT at baseline was likewise poor; while the reference equation for a healthy counterpart predicted a distance of 555.3 m^14^, the initial mean distance was only 67 m which evolved to 124 m at follow-up. These baseline measures underscore the initial low functional performance of the study participants and highlight the need for longer or more intensive interventions to achieve significant improvements in all areas of physical function.

Low muscle strength in brain tumor patients may stem from various factors, including neurologic deficit, muscle disuse, nutritional deficit, or medication related to anti-cancer therapy such as steroids. While the standardized cut-offs for defining low muscle strength have not yet been established within the definition of tumor-associated cachexia and sarcopenia^39^, each different cause may necessitate unique criteria for nutritional intervention. This study underscores the clinical importance of protein supplementation in patients without sarcopenia in need of significant functional gain.

The significant correlations in the protein group between enhanced balance, functional independence, muscle mass, strength, and mobility suggest that these improvements could be attributed to protein supplementation. The average baseline score of the BDI among all participants was 15, which is suggestive of mild depression^40^. Similarly, an average BFI score of 4 suggests fatigue that, while noticeable, does not overwhelmingly affect daily activities. The lack of differences in BDI and BFI scores between groups could be linked to the initially mild symptoms in both groups. Moreover, due to the brain lesion-related disabilities, participants may have felt dissatisfied with their health compared to their pre-cancer condition.

There are some limitations of this study. One is single institution-based participant recruitment, which resulted in limited number of participants and rather heterogeneous group of brain tumors with variable prognosis (glioblastoma 63.6%). Future multicenter studies with more patients will provide more specific information for each type of brain tumor. Also, the study had relatively low number of eligible candidates (50.8% screened) by excluding those with cardiac comorbidities and diabetes to avoid bias; the participants in this study may not represent those with such comorbidities. Another limitation is the 6-week duration of intervention which may have been too short to bring about statistical differences between the protein and control groups in all of assessments. In addition, the study had relatively low participation rate (68%) probably due to the difficulties during tumor treatment, yet those who agreed to participate had relatively low drop-out rate (27%). This also may have contributed to the disproportion of malnutrition status of participants in which no severely malnourished participants were observed. Additive to the immediate effect of the protein supplementation intervention in this study, the effect-lasting duration of intervention and the duration needed for the most efficient results still need to be determined. Further studies with stratified causes would enable individualized nutritional support with the best efficiency.

In conclusion, this study demonstrated that a 6-week regimen of protein supplementation significantly enhanced rehabilitative potential in brain tumor patients via improvements in inflammatory markers, nutritional status, muscle strength, and gait function. Despite the brief duration of the intervention, its effects on rehabilitation outcomes were particularly significant in malnourished individuals. The use of protein supplements was safe and feasible in this patient population. Protein supplementation could lower inflammatory potential, enhance physical fitness and function, and may further extend overall survival in brain tumor patients. Further research with a larger cohort is needed to determine the optimal duration and amount to sustain these secondary function gains.

## Data Availability

The datasets used during the current study are available from the corresponding author upon reasonable request.
